# Classification of Thyroid Tumors Based on Mass Spectrometry Imaging of Tissue Microarrays; a Single-Pixel Approach

**DOI:** 10.3390/ijms21176289

**Published:** 2020-08-31

**Authors:** Agata Kurczyk, Marta Gawin, Mykola Chekan, Agata Wilk, Krzysztof Łakomiec, Grzegorz Mrukwa, Katarzyna Frątczak, Joanna Polanska, Krzysztof Fujarewicz, Monika Pietrowska, Piotr Widlak

**Affiliations:** 1 Maria Skłodowska-Curie National Research Institute of Oncology, Gliwice Branch, 44-102 Gliwice, Poland; agata.kurczyk@io.gliwice.pl (A.K.); marta.gawin@io.gliwice.pl (M.G.); mykola.chekan@io.gliwice.pl (M.C.); monika.pietrowska@io.gliwice.pl (M.P.); 2 Faculty of Automatic Control, Electronics and Computer Science, Silesian University of Technology, 44-100 Gliwice, Poland; agatamkwilk@gmail.com (A.W.); krzysztof.lakomiec@polsl.pl (K.Ł.); grzegorz.mrukwa@polsl.pl (G.M.); katarzyna.fratczak@polsl.pl (K.F.); joanna.polanska@polsl.pl (J.P.); krzysztof.fujarewicz@polsl.pl (K.F.)

**Keywords:** thyroid cancer, molecular classifiers, molecular imaging, bioinformatics, proteomics, mass spectrometry imaging, biomarkers

## Abstract

The primary diagnosis of thyroid tumors based on histopathological patterns can be ambiguous in some cases, so proper classification of thyroid diseases might be improved if molecular biomarkers support cytological and histological assessment. In this work, tissue microarrays representative for major types of thyroid malignancies—papillary thyroid cancer (classical and follicular variant), follicular thyroid cancer, anaplastic thyroid cancer, and medullary thyroid cancer—and benign thyroid follicular adenoma and normal thyroid were analyzed by mass spectrometry imaging (MSI), and then different computation approaches were implemented to test the suitability of the registered profiles of tryptic peptides for tumor classification. Molecular similarity among all seven types of thyroid specimens was estimated, and multicomponent classifiers were built for sample classification using individual MSI spectra that corresponded to small clusters of cells. Moreover, MSI components showing the most significant differences in abundance between the compared types of tissues detected and their putative identity were established by annotation with fragments of proteins identified by liquid chromatography-tandem mass spectrometry in corresponding tissue lysates. In general, high accuracy of sample classification was associated with low inter-tissue similarity index and a high number of components with significant differences in abundance between the tissues. Particularly, high molecular similarity was noted between three types of tumors with follicular morphology (adenoma, follicular cancer, and follicular variant of papillary cancer), whose differentiation represented the major classification problem in our dataset. However, low level of the intra-tissue heterogeneity increased the accuracy of classification despite high inter-tissue similarity (which was exemplified by normal thyroid and benign adenoma). We compared classifiers based on all detected MSI components (*n* = 1536) and the subset of the most abundant components (*n* = 147). Despite relatively higher contribution of components with significantly different abundance and lower overall inter-tissue similarity in the latter case, the precision of classification was generally higher using all MSI components. Moreover, the classification model based on individual spectra (a single-pixel approach) outperformed the model based on mean spectra of tissue cores. Our result confirmed the high feasibility of MSI-based approaches to multi-class detection of cancer types and proved the good performance of sample classification based on individual spectra (molecular image pixels) that overcame problems related to small amounts of heterogeneous material, which limit the applicability of classical proteomics.

## 1. Introduction

Though thyroid nodules are very common in the overall population, malignant tumors occur in less than 1% of such nodules. Nevertheless, thyroid cancer is the most common endocrine cancer and contributes to 1–2% of all new malignancies diagnosed each year worldwide. The majority of thyroid carcinomas originate from follicular epithelial cells and include well-differentiated papillary thyroid carcinomas (PTC; up to 80% of all thyroid malignancies) and follicular thyroid carcinomas (FTC; about 15% of all thyroid malignancies). Moreover, undifferentiated anaplastic thyroid carcinoma (ATC; 1–2% of all thyroid cancers), which is the most aggressive thyroid malignancy, also develop from epithelial cells. Additionally, medullary thyroid carcinoma (MTC) is derived from the neuroendocrine parafollicular C-cell comprises 3–5% of all thyroid cancers [1,2]. Currently, most of the thyroid tumors are diagnosed by pathomorphological assessment alone, and such classification is the primary step in the assessment of prognosis and selection of the treatment [3]. The primary diagnosis in the majority of patients with thyroid cancers is based on fine-needle aspiration cytology (FNAC) of thyroid nodules; then, further diagnosis is performed based on histopathological intra- or post-operative examination of the resected thyroid tissue [4,5]. However, in some cases, cytological and histological patterns are ambiguous, and proper classification is problematic [6]. Therefore, the classification of thyroid tumors, particularly those exhibiting unusual morphological patterns, might be improved if additional molecular tests could be applied.

It has been generally accepted that new biomarkers identified with the use of high-throughput “omics” approaches could markedly support the classification of thyroid cancers based on histopathological patterns [7,8,9]. However, one should be aware that the initial diagnosis of thyroid tumors is based on FNAC; hence, hypothetical biomarkers have to be compatible with cytological material collected by fine needle biopsy. Therefore, the major obstruction associated with such a biospecimen is a low amount of material (usually up to a few hundred cells) and its high heterogeneity (usually a mixture of tumor cells and other types of cells). It has been proposed that challenges associated with the molecular classification of thyroid cancer using cytological material could be approached by mass spectrometry imaging (MSI). It is an emerging technology in biomedical research and has been recently evolving into a powerful tool in the study of various types of diseases. The major benefit of MSI is the possibility of combining molecular and morphological information. Molecular images generated by mass spectrometry are spatially resolved and correlated with the respective histological images; hence, molecular profiles could be allocated to specific cells or tissue regions. Moreover, different molecular species, e.g., proteins, peptides, lipids, drugs, and their metabolites, can be imaged, significantly broadening the amount of information derived from a tissue [10,11,12,13]. Among many applications of MSI, there was molecular characterization and classification of different types of solid tumors, including thyroid cancers [14,15,16]. It might be expected that the limitations of fine needle biopsy–derived material could be overcome by MSI due to its ability to provide information collected from a low number of specific cells present in cytological samples. The idea that MSI is capable of distinguishing between different papillary tumors using proteomic signatures of cytological FNA samples has been validated experimentally in a series of reports from Fabio Pagni and Fulvio Magni laboratories who used a Matrix-Assisted Laser Desorption/Ionization Time-of-Flight (MALDI-TOF) MSI approach [17,18,19]. Moreover, metabolome profiling by Desorption Electrospray Ionization (DESI) MSI was also recently tested for the diagnosis of thyroid tumors based on FNA samples [20].

Molecular classification of cancer that is based on MSI analysis of cytological and histological samples has several challenges related to the implemented methodology both at (pre)analytical [21,22] and data processing [23,24] steps. The latter step involves the necessity to solve several problems related to the processing of heterogeneous samples and optimization of feature selection for cancer classifiers. Here, we implemented several biostatistical approaches to test the suitability of spectral data generated by MALDI-TOF MSI for multi-class comparison and classification of different thyroid tumors. Tissue microarrays were used as a surrogate of cytological smears assuming a comparable number of spectra registered by MSI in both types of biospecimens. The classification of thyroid tumors using tissue microarrays analyzed by MALDI-TOF MSI was previously reported by Galli et al. [25], who compared material from benign tumors (follicular adenoma and hyperplastic lesions) and papillary thyroid cancer (both classical and follicular variant). However, mean spectra for tissue cores were used in that case. In the present work, the assessment of similarities and differences between seven types of thyroid specimens was based on features of individual spectra (i.e., pixels of molecular images), which seems to be a more relevant approach to potential applications in molecular diagnostics of cancer.

## 2. Results

**Figure 1:**
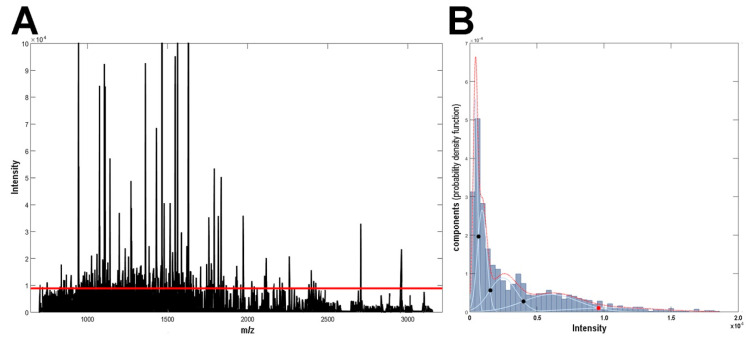
Mass spectrometry imaging (MSI) spectrum. (**A**) The average mass spectrum. (**B**) Distribution of m/z components with different average intensity; the red dotted curve represents an intensity distribution model with its constituents represented by blue lines (dots represent thresholds delineating intensity-related classes of components). The red line in Panel A represents the threshold separating 147 most abundant components (>9610 a.u.).

Tissue specimens representative for seven types of thyroid tissue (ROIs) were analyzed by MALDI-MSI. There were about 400 tissue cores analyzed derived from 134 patients with either follicular adenoma (FA), classical or follicular variant of papillary thyroid carcinoma (PTC-CV and PTC-FV, respectively), follicular thyroid carcinoma (FTC), anaplastic thyroid carcinoma (ATC), or medullary thyroid carcinoma (MTC); normal thyroid (NT) samples were collected from tissue region showing normal thyroid histology. About 135,000 spectra were registered (360 spectra per one tissue core, on average); the general characteristics of the analyzed biomaterial are presented in Supplementary Table S1. Mean spectra obtained for each type of ROI are presented in Supplementary Figure S1. Gaussian mixture modeling was used for the identification of spectral components that resulted in 1536 components that corresponded to tryptic peptides; the averaged spectrum used for the model construction is depicted in Figure 1. Importantly, this approach provided a substantial reduction of data dimensionality, which reduced the computation load for the processing of multiple spectra.

**Figure 2:**
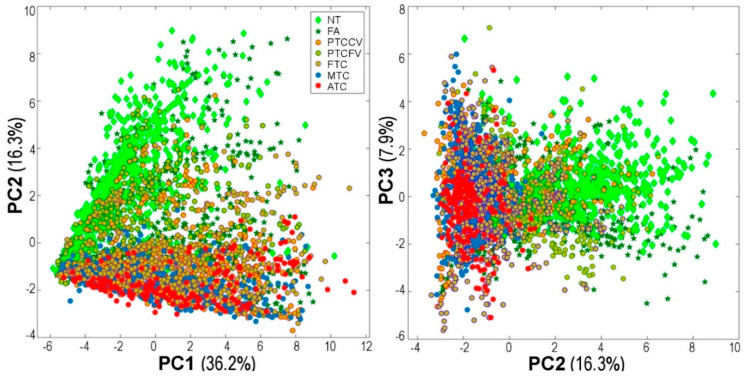
Principal component analysis (PCA) of spectra representative for all regions of interest (ROIs). Ten spectra were randomly selected from each tissue core for clarity; here, three principal components together describe 60% of the variability.

In the first step, general similarities between spectra derived from different types of ROIs were estimated. The principal component analysis revealed a clear separation of spectra representative for normal thyroid and spectra representative for ATC, MTC, and the majority of well-differentiated thyroid cancers (WDTC, including FTC and both variants of PTC); the most differentiating for normal and cancer spectra was PC2 responsible for 16.3% of the general variability (Figure 2) (PC1 was putatively associated with inter-individual differences present within each patients’ subset). However, spectra representative for FA overlapped with both normal thyroid and cancer ROIs. Spectra representative for WDTC, ATC, and MTC could not be separated reasonably along with any principal component (Figure 2).

**Figure 3:**
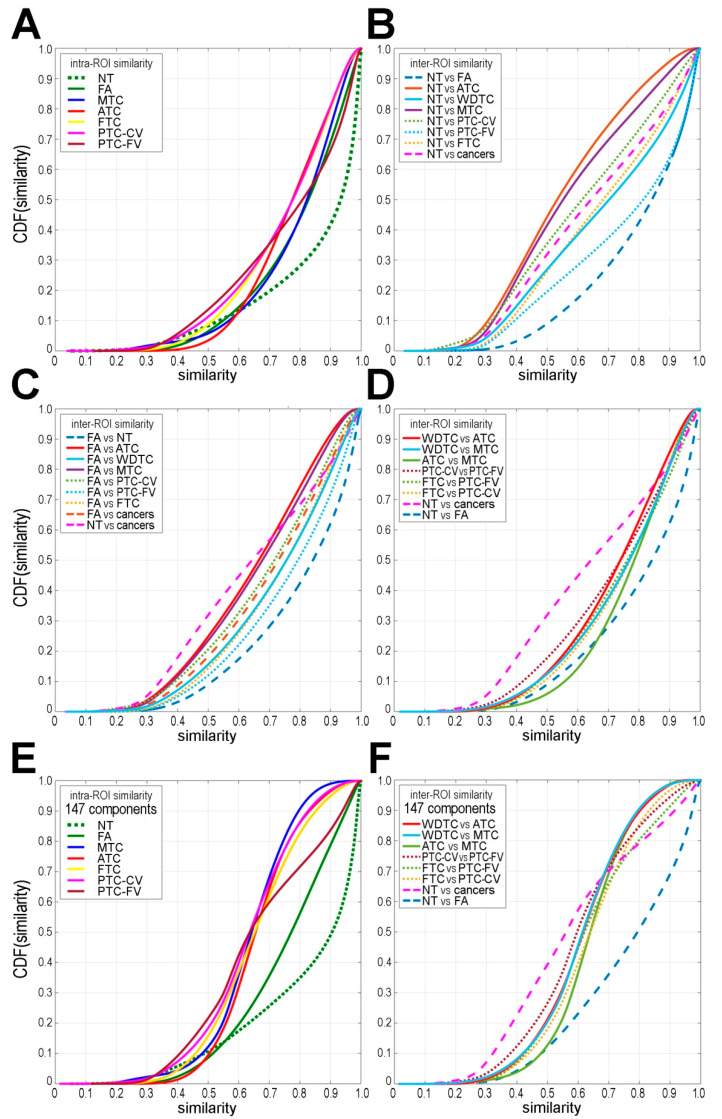
Molecular similarity among different types of thyroid tissue. Depicted are the cumulative distribution functions of similarity (cumulative distribution function (CDF(similarity)), either intra-ROI (panel **A**) or inter-ROI (panels **B**–**D**) when all 1536 components were considered. Intra-ROI (panel **E**) and inter-cancer ROI (panel **F**) similarity was based on 147 most abundant components. A similarity that corresponded to CDF(similarity) = 0.5 represents its median value.

Next, the similarity index between pairwise compared spectra from seven types of ROIs was estimated based on all registered molecular components (i.e., 1536 m/z components) to address tissue heterogeneity; the resulting cumulative distribution functions (CDFs) of similarity are depicted in Figure 3A–D. To assess intra-tissue heterogeneity, similarities between spectra from the same type of ROI were compared (Figure 3A). We found the highest homogeneity for normal thyroid tissue (median similarity of 0.93) and the highest heterogeneity for ATC (median similarity of 0.77); even higher heterogeneity (i.e., lower intra-ROI similarity) was observed if spectra from all cancer ROIs (or all WDTC) were combined (Supplementary Table S2). To assess inter-tissue heterogeneity, similarities between spectra from different ROIs were compared (Supplementary Table S3). When normal thyroid was compared with different thyroid tumors, the highest similarity was observed between NT and benign FA (median similarity of 0.85) and the lowest similarity was observed between NT and the undifferentiated ATC (median similarity of 0.53), while similarities between NT and differentiated cancers were flanked by these two extremes (Figure 3B). When benign FA was compared with malignant tumors, the highest similarity was observed between FA and follicular variant of PTC and FTC (median similarity of 0.79 and 0.75, respectively), while the lowest similarity was observed between FA and ATC (median similarity of 0.66) (Figure 3C). Median similarities between different types of malignant tumors were in the range between 0.79 and 0.69. Similarities between three major types of thyroid cancer were comparable—the median similarities between WDTC vs. ATC, WDTC vs. MTC, and ATC vs. MTC were 0.74, 0.77, and 0.79, respectively (Figure 3D).

A similar analysis was performed taking into account 147 most abundant components (Figure 1). In this case, high intra-ROI similarity within NT and FA remained (median similarity of 0.91 and 0.78), yet intra-cancers similarities were markedly reduced (median similarities in the range between 0.65 and 0.62; Figure 3E, Supplementary Table S2). Furthermore, although inter-ROI similarity between NT and FA remained high (median similarity of 0.80), similarities among other types of ROI were markedly reduced when the 147 most abundant components were considered (Supplementary Table S3). Noteworthy, inter-ROI similarities between different types of cancer were comparable (median similarity in the range between 0.62 and 0.58; Figure 3F). These observations suggested collectively that components with a lower intensity markedly contributed to similarities among spectra, while a significant differential potential could be attributed to highly abundant components (with the particular exception of the similarity index between NT and FA).

**Figure 4:**
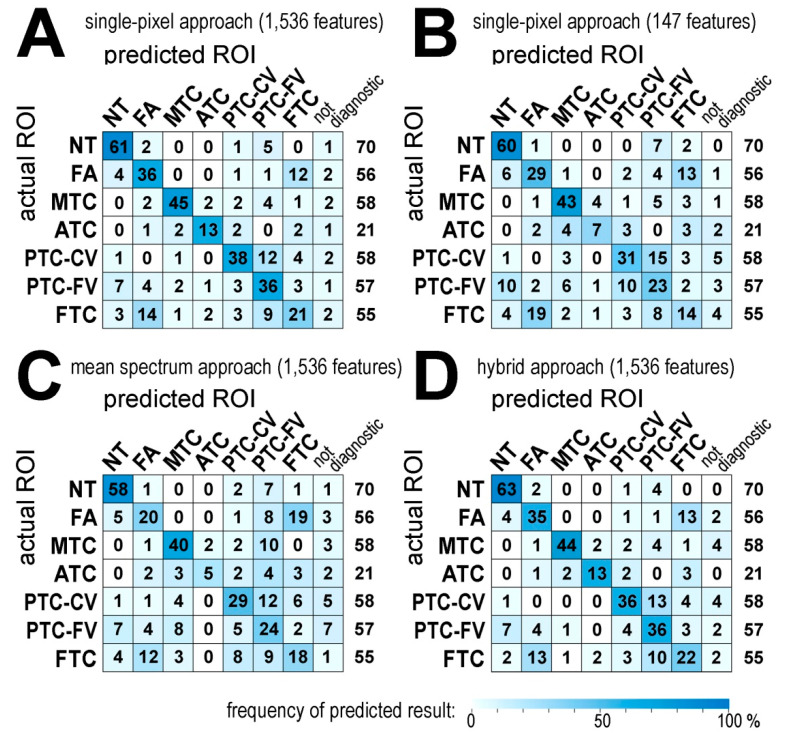
Classification of normal and cancerous thyroid tissue cores based on MSI-registered spectra. Results of a core class prediction are presented as actual numbers (a relative frequency of specific results is color-coded according to the color scale); the last column represents the total number of cores. Presented are: a single-pixel approach for 1536 features (panel **A**) and 147 features (panel **B**), a mean spectrum approach for 1536 features (panel **C**), and a hybrid approach for 1,536 features (panel **D**).

In the next step, the classification of tissue cores was performed based on the classification of individual spectra that we called “a single-pixel approach.” First, binary classification models were tested for comparison of spectra from all possible pairs of seven ROIs (i.e., 21 classifiers were tested); then, individual spectra in a tissue core were analyzed using these multiple models. Classifier features involved either the complete set of components (*n* = 1536) or the subset of the most abundant components (*n* = 147). In the former case, the accuracy of binary classifiers was in the range between 0.94 (NT vs. ATC) and 0.63 (FA vs. FTC) with the average 0.84; in the latter case, binary classifiers performed comparably (average accuracy 0.81) (Supplementary Table S4). Subsequently, all individual spectra in all tissue cores were analyzed using all 21 binary classifiers, and then an individual spectrum was considered “classified” only if all 6 binary classifiers including a given ROI showed full concordance. Otherwise, a spectrum was considered “not classified”, which provided a high level of confidence in the individual spectrum classification. In general, 7% and 10% of spectra remained not classified when classifiers based 1536 and 147 components were applied, respectively (Supplementary Table S4). The largest and the lowest number of not classified spectra was observed for ATC and NT cores (13% and 3%, respectively, on average), which mirrored the level of intra-ROI heterogeneity established earlier using the similarity index. Finally, tissue cores were classified based on the predicted identity of individual spectra: a core was classified according to the most frequent class in corresponding spectra (including the “not classified” type). In general, the precision of such core classification was higher for spectra classifiers based on all 1536 components than for classifiers based on the most abundant components (67% and 55% of cores were classified properly, respectively) despite overall similar accuracy of binary classifiers. Nevertheless, in both cases, the overall correctness was much higher than approx. 14% expected from random indexing of seven classes. The complete table of classification results is presented in Figure 4A,B. The lowest accuracy of classification was observed for FTC that was frequently confused with FA and PTC-FV (the sensitivity of FTC classification was 0.38 and 0.26 for classifiers based on 1536 and 147 components, respectively). Moreover, low sensitivity (0.33) was observed in the classification of ATC using classifiers based on 147 components. Furthermore, the number of not classified (i.e., “not diagnostic”) cores was higher when the classification was based on the most abundant components (16 vs. 11 for classification based on 147 and 1536 components, respectively). Hence, we concluded that the classification of tissue cores performed better using models based on all registered MSI components. Furthermore, we also tested two other classification strategies based on mean spectra computed for separate tissue cores. First, binary classification models were tested using mean spectra, and then cores were classified based on their mean spectra (“a mean spectrum approach”). In this case, binary classifiers performed comparably to binary classifiers based on individual spectra (average accuracy 0.82 for models based on 1536 MSI components; Supplementary Table S4). However, the performance of a final core classification was much lower than that of a single-pixel approach—only 52% of cores were classified correctly (Figure 4C). Second, binary classification models tested using individual spectra were applied for classification of cores based on their mean spectra (“a hybrid approach”). In this case, the overall precision of a final core classification was only slightly lower compared to a single-pixel approach: 66% of cores were classified correctly (Figure 4D). Notably, cores corresponding to the most homogenous normal thyroid showed even higher precision of classification using this hybrid approach (63/70 vs. 61/70 cores predicted properly). Nevertheless, the number of “not diagnostic” cores was higher when a hybrid approach was implemented (2.9%, 3.7%, and 5.9% of not classified cores using a single-pixel, a hybrid, and a mean spectrum approach, respectively). Hence, we concluded that a single-pixel approach performed best for the classification of analyzed tissue cores.

**Figure 5:**
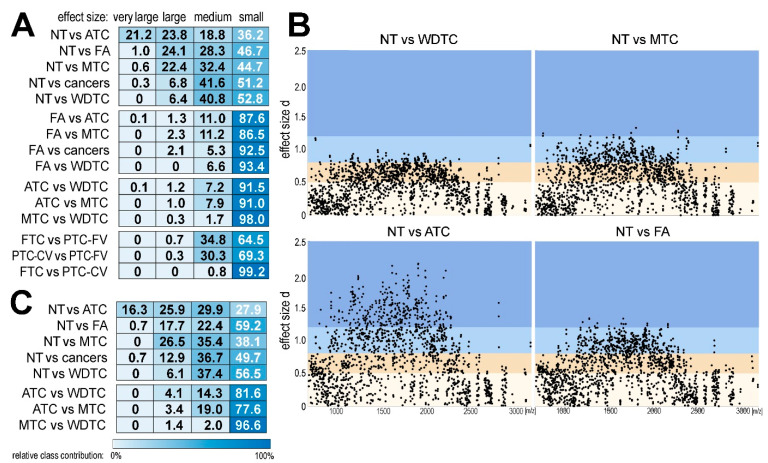
Molecular components that discriminated different types of thyroid tissue. (Panel **A**)—relative contribution (percentage) of components that differentiated pairwise selected ROIs with very large, large, medium, or small effect sizes (all detected components analyzed; *n* = 1536). (Panel **B**)—distribution of discriminatory components in the m/z axes in four selected pairwise ROI comparisons; very large (>1.2), large (>0.8), and medium (>0.5) effect sized are color-coded (dark blue, light blue, and beige, respectively); each dot corresponds to one spectral component. (Panel **C**)—relative contribution (percentage) of the most abundant components (*n* = 147) that differentiated pairwise selected ROIs with very large, large, medium, or small effect sizes.

In the last step, molecular components with markedly different abundance between types of thyroid specimens were detected (all spectra from a given ROI were combined for this analysis). Considering the structure of data, the strength of differences was estimated by the effect size factor; Cohen’s *d* (absolute) values above 0.5, 0.8, and 1.2 corresponded to medium, large, and very large effects, respectively [26]. The number of components that discriminated pairwise against different ROIs with assumed effect sizes is illustrated in Figure 5 (see details in Supplementary Table S5). First, discriminatory components were detected in the complete set of 1536 molecular components. A substantial number of components with significantly different abundances were detected between normal thyroid and thyroid tumors. The highest number of discriminatory components was observed between NT and ATC (45% of all components showed large or very large effect size). There were fewer discriminatory components between NT and MTC (23% of components showed large or very large effect size), while the number of components markedly discriminating NT from WDTC was relatively low (about 6% of the registered components showed large effect size). Surprisingly, despite a very high level of general similarity between NT and FA, there was a large number of components whose abundances were markedly different between these two types of ROI, which putatively reflected high homogeneity (i.e., low variability) inside both tissues. On the other hand, there were much fewer components whose abundance was markedly different between benign FA and malignant cancers; notably, components discriminating between FA and WDTC showed only medium effect size. When three major types of thyroid cancer were compared (WDTC, ATC, and MTC), a higher number of components showing large/very large effect was noted between undifferentiated ATC and differentiated cancers (WDTC and MTC). Unexpectedly, we found that the number of discriminatory components between FTC and PTC-CV was lower than the number of discriminatory components between FTC and PTC-FV or between both variants of PTC (Figure 5A). Mass distribution of components discriminating selected types of ROI is depicted in Figure 5B. Moreover, the detection of discriminatory components was performed using 147 most abundant m/z components (Figure 5C). The relative proportion of differentiating components (medium, large, and very large effect size) was similar or even higher in this subset of very abundant components when compared to the whole set of 1536 components (with a marked exception of the reduced contribution of significant differences between NT and FA).

The hypothetical identity of MSI components was established by attributing masses (m/z values) of imaged molecular components (i.e., tryptic peptides) to measured masses of peptides identified by liquid chromatography-tandem mass spectrometry (LC-MS/MS) in tissue lysates. However, though hypothetical identity was attributed to the majority of molecular components detected by MSI, one should be aware that this type of annotation is not unique and more than one identified peptide could be matched to an MSI component due to relatively low resolution of MALDI-TOF MSI (Supplementary Tables S6–S8). Nevertheless, proteins whose tryptic fragments were most frequently attributed to MSI components markedly upregulated in all types of tumor ROIs (compared to normal thyroid) included thyroglobulin, heat shock proteins, and proteins associated with several gene ontology (GO) terms involved in cancer development: response to growth factors, cytoskeleton organization, extracellular matrix organization, cell-cell adhesion, cell motility, glycolysis and glucose metabolism, regulation of immune response, and inflammatory response (Supplementary Table S9). Moreover, proteins whose tryptic fragments were attributed to MSI components specifically upregulated in MTCs included those involved in their neuroendocrine functions (calcitonin and chromogranin A), and other proteins showed previously to be specifically upregulated in MTC (e.g., APOE, CEACAM5, TTR) [27]. Furthermore, proteins whose tryptic fragments were attributed to MSI components specifically upregulated in ATC included those associated with GO terms involved in cancer progression: regulation of cell migration, regulation of angiogenesis, cytoskeleton organization, regulation of cell death, cell-cell adhesion, and regulation of immune and inflammatory response (Supplementary Table S9). On the other hand, very few components specifically distinguished benign adenoma: when compared to normal thyroid all components upregulated in FA were also upregulated in malignant cancers, while only two components (m/z 1183.6 and 1184.6) markedly upregulated in cancer (large or very large effect size) were not upregulated in FA (small effect size) (components putatively corresponded to a fragment of ribonucleoprotein HNRNPUL2). Furthermore, no components showed marked differences (large or very large effect size) between FA and well-differentiated cancers.

## 3. Discussion

Classification of thyroid tumors based on pathomorphological features is the primary step in the assessment of prognosis and selection of the treatment. The majority of patients are diagnosed based on the fine needle aspiration cytology (FNAC) of thyroid nodules with further validation and verification based on histopathological examination (either intra- or post-operative) of the resected tissue. Unfortunately, in some cases, cytological and histological patterns are ambiguous, and proper classification is problematic [3,4,5,6,28,29]. For example, thyroid tumors with follicular growth pattern include a broad range of lesions that are difficult to distinguish by cytology and could be challenging even in histologic specimens. These lesions include hyperplastic nodules, benign follicular thyroid adenomas (FAs), follicular carcinomas (FTCs), and follicular variant of papillary thyroid carcinomas (PTC-FVs). On the other hand, the most common PTC shares some cytological features with benign lesions (nodular hyperplasia, FTA, Hashimoto’s thyroiditis) as well as other malignant lesions (e.g., Hürthle cell carcinoma, FTC, and MTC) [29,30,31]. Hence, supporting the assessment of morphological patterns with molecular biomarkers would be recommended to improve the classification of thyroid tumors [7,9]. Assuming that the primary diagnosis of thyroid disease is usually based on the fine needle biopsy of thyroid nodules, potential molecular tests would be preferably performed using this type of specimen. However, a small amount of heterogeneous material present in such a biopsy represents a diagnostic challenge for “classical” methods of genomics and proteomics typically based on tissue lysates. A few reports proved already the concept that mass spectrometry imaging could be implemented in the molecular classification of thyroid cancer to overcome these limitations of cytological material [17,18,19,20,21,22]. Importantly, individual spectra/pixels registered by MSI could be used for sample classification. However, this requires specific approaches to the optimization of data processing [23,24].

Here, tissue microarrays representative for five major types of thyroid malignancies (medullary cancer, undifferentiated anaplastic cancer, and three types of well-differentiated cancers); benign thyroid tumor (adenoma); and normal not cancerous thyroid tissue were analyzed by MALDI-MSI then individual “pixels” of MSI images were used for assessment of differences between tissue types and sample classification to mimic the situation typical for cytological smears. Different numerical approaches were tested to assess molecular similarity within and between types of thyroid tissue (i.e., specific ROIs). The unsupervised PCA approach revealed a large intra- and inter-ROI variability of MSI spectra. Though some separation of normal thyroid from all types of malignancies was visible, specific types of cancers cannot be separated and benign adenoma overlapped with both normal and cancerous tissue, which defined the major classification problem. To estimate overall intra- and inter-ROIs heterogeneity more specifically, the similarity index was calculated between individual spectra based on the spectral contrast angle approach, which previously proved its high applicability in the analysis of MS data [32]. We found the lowest and the highest intra-ROI heterogeneity for normal thyroid and undifferentiated ATC, respectively, which further affected the performance of the classification of these types of tissue. As expected, normal thyroid was more similar to benign FA than to malignant cancers and more similar to well-differentiated epithelial cancers than to ATC or MTC. Importantly, FA was very similar to well-differentiated cancers with a follicular morphology (FTC and PTC-FV), whose proper discrimination represents the major diagnostic challenge also in the case of classical pathological assessment. The multi-class problem of separation of thyroid tumors was further tested using classification models. Assuming seven classes of thyroid tissue, 21 SVM-based one-versus-one binary classifiers were built to cover all possible combinations of ROIs, then each spectrum was tested with all of them (noteworthy, this strategy outperformed typical multiclass model based on the KNN approach; data not presented). In general, the accuracy of binary classifiers reflected overall similarity between specific ROIs, being high for models comparing normal thyroid with malignant cancers and low for models comparing well-differentiated cancers. An interesting exemption was a high accuracy of the model for the classification of normal thyroid vs. benign adenoma, which likely reflected a high level of intra-ROI homogeneity of both specimens. The discrimination between FA and FTC represented the largest classification problem (discrimination of FA from both variants of PTC performed better). Nevertheless, an individual spectrum was considered “classified” only if decisions were univocal, i.e., all six models featuring a particular type indicated that class; otherwise it remained not classified (“not diagnostic”), which increased credibility of the class prediction. To verify the actual reliability of such classification, the resulting classes of individual spectra were used for the tissue core classification. Importantly, 2/3 of cores were classified properly, while random indexing of seven classes would result in a proper assignment of 14% of samples only. The best performance was observed in the case of normal thyroid and MTC (about 80% of cores classified properly), while the worst performance was observed for FTC (only 38% of cores were classified properly, while 25% were confused with FA). The performance of the classification depended not only on similarities/differences between compared tissues per se but also on the level of intra-tissue heterogeneity. Therefore, undifferentiated ATC that showed very high intra-tumor heterogeneity could represent a classification problem (38% of false-negatives) despite its low overall similarity to other types of thyroid tissue. Additionally, we searched for spectral components that showed the most significant differences in abundance between pairwise compared ROIs using the effect size approach. As expected, a high contribution of differentiating components was usually reversibly associated with the overall similarity and increased accuracy of sample classification. However, a few interesting exemptions from this general rule were noted as mentioned above, which could be explained by the influence of the intra-tissue heterogeneity. Hence, the estimation of this parameter should be always recommended when a comparison between different tissue types is planned. Identification of differentially expressed MSI components based on matching their masses with masses of peptides detected by the classical LC-MS/MS in corresponding tissue lysates is only putative and should be used with caution. Nevertheless, the hypothetical identity of differentiating MSI components fitted general proteome profiles of different thyroid diseases established by classical MS-based proteomics approaches [27], which confirmed the credibility of the tumor classification approach proposed in the current work.

In our basic approach, all molecular components present in MSI data (i.e., 1536 spectral components corresponding to tryptic fragments of proteins) were used in analyses without any preselection. However, we also tested a subset of the 147 most abundant components (putatively corresponding to fragments of more abundant proteins). Interestingly, we found that the overall similarity between the compared tissues was lower when the estimation was based on the latter subset. Similarly, the relative contribution of components showing (very) large effect size of differences between tissue types was generally higher in the subset of the most abundant ones. Hence, one could conclude that effective differentiation of tissue types could be performed using highly abundant components only which are less sensitive to analytical errors. However, we observed that molecular classifiers based on all components (i.e., including low abundance components) generally performed better than classifiers based on the most abundant components only; hence, the utilization of all registered MSI components for sample classification appeared a credible solution. Artificial intelligence-based methods for the preselection of features for molecular classifiers are frequently in use [33], which approach was validated for MSI-based classification of cytological smears from thyroid tumors [23]. However, a selection of the “best set” of components for sample classification based on the machine learning approaches could be sensitive to specific features present uniquely in a given dataset, which implies the necessity of using “big data” (i.e., very large patients cohorts or sample repositories). Still, we tested here classification models based on spectral components selected using t-test results between ROIs, yet this approach did not improve the performance of classification compared to models based on all components (data not presented). Therefore, using all registered MSI components for the classification based on individual spectra remains a valid alternative when computationally feasible. Nevertheless, using either all components or their pre-selected subset emphasizes the problem of proper spectra generation and data processing to avoid errors and artifacts potentially related to the analysis of low-signal components of MSI spectra. Moreover, we found that a single-pixel approach outperformed a classification model based on the mean spectra of tissue cores. Alternatively, a strategy where binary classification models tested with individual spectra are subsequently used for the sample classification based on its mean spectrum could be considered, yet the implementation of this hybrid approach should be limited to tissue specimens with high homogeneity.

## 4. Materials and Methods

### 4.1. Clinical Material

Postoperative tissue was collected during thyroidectomy then stored as formalin-fixed paraffin-embedded (FFPE) material. Samples derived from 134 patients treated at Maria Skłodowska-Curie National Research Institute of Oncology in Gliwice between 2012–2017. The study was approved by the appropriate local Ethics Committee (approval no. KB/430–17/13). Tissue blocks were used to generate tissue microarrays (TMAs) that included 375 individual cores (3 cores from different tissue areas per patient, on average) of 1 mm in diameter. Tissue cores represented seven types of thyroid tissue (Region of Interest, ROI): normal thyroid (NT), follicular adenoma (FA), papillary thyroid carcinoma—classical variant (PTC-CV), papillary thyroid carcinoma—follicular variant (PTC-FV), follicular thyroid carcinoma (FTC), anaplastic thyroid carcinoma (ATC), and medullary thyroid carcinoma (MTC). Normal thyroid was represented by a tissue distant from the cancer region that showed no marks of any pathology. Clinical material included in the study is characterized in the Supplementary Table S1. Tissue cores were distributed randomly into eight arrays, and molecular images were registered in a random sequence.

### 4.2. Sample Preparation for MALDI-MSI

Tissue microarray blocks were cut into 5 µm sections with the use of a rotary microtome (HM 340E, Thermo Fisher Scientific, Waltham, MA, USA) and placed on ITO glass slides (Bruker Daltonik, Bremen, Germany) coated with poly-l-lysine. Slides were then dried at 37 °C for 18 h. Additional thermal treatment (60 °C, 1 h) was performed directly before paraffin removal. De-waxing was carried out in xylene (2 × 5 min), followed by washing with 99.8% ethanol (5 min), 96% ethanol (5 min), and 50% ethanol (5 min). Finally, glass slides were left to dry on a bench and subjected to heat-induced antigen retrieval in 10 mM Tris-HCl pH 9.0, 95 °C, 20 min (in a water bath). The slides were cooled in the retrieval solution for 20 min at room temperature, then washed with MilliQ water for 1 min and dried on a bench for 10 min, followed by drying in a vacuum desiccator for 15 min. A solution of sequencing grade modified trypsin (Promega, Madison, WI, USA) (20 µg/mL in 25 mM NH_4_HCO_3_) was sprayed over each TMA section with the use of SunCollect device (SunChrom, Friedrichsdorf, Germany). The section was then incubated in a humid chamber with MilliQ water for 18 h at 37 °C. After on-tissue digestion, the slide was dried in a vacuum desiccator for 15 min and covered with an α-cyano-4-hydroxycinnamic acid (5 mg/mL w 50% ACN, 0.3% TFA) matrix solution deposited onto TMAs with the use of SunCollect device. Methods of both trypsin and matrix coating were employed according to the reference [34].

### 4.3. MALDI-MSI Measurements

Spectra of tryptic peptides were acquired using MALDI-TOF mass spectrometry with the use of ultrafleXtreme mass spectrometer (Bruker Daltonik, Bremen, Germany) equipped with smartbeam II™ laser operated at 1 kHz frequency. Ions were accelerated at 25 kV with a PIE time delay of 100 ns. Spectra were recorded in reflectron positive mode within 700–3700 m/z, 400 shots per position, the random walk mode was activated (40 shots at raster spot). Each TMA core was imaged with a raster width of 50 µm (laser setting: 3_medium). After imaging, the matrix was washed off the glass slides with 70% ethanol (two washes, 1 min each), and the sections were stained with hematoxylin and eosin, then scanned and used for image co-registration (using flexImaging software; Bruker Daltonik, Bremen, Germany). Compass for flex 1.4 software package (Bruker Daltonik, Bremen, Germany) was used for spectra acquisition and handling.

### 4.4. Spectra Processing and Identification of Spectral Components

The MSI spectra dataset was preprocessed by performing mass channels unification, baseline subtraction [35], outlying spectra identification according to TIC (total ion current) value using criterion for skewed and heavy-tailed distributions [36], fast Fourier transform-based peak alignment to reference average spectrum [37], and TIC normalization. Gaussian mixture modeling (GMM) of the average spectrum was applied for peak detection as described in detail elsewhere [38,39]. GMM components of low amplitude and high variance were removed from initial GMM spectra representation; GMM components modeling the same spectrum peak were merged by summing their estimated abundance and setting the location of a dominant component as mass/charge value of a peptide ion. The abundance of the particular component was estimated by pairwise convolution of the GMM components and individual spectra. The resulting dataset featured 1536 components detected in mass/charge range between 700 and 3150 that represent tryptic peptide species imaged by MSI. The average intensities of these 1536 components ranged between 25 and 830,527 arbitrary units, whose distribution could be described by five Gaussian components with the following thresholds: >25, >639, >1507, >4001, and >9610 a.u.; this resulted in five classes, with 386, 328, 332, 343, and 147 (the most abundant) m/z components, respectively (see Figure 1B).

### 4.5. Statistical Analyses and Sample Classification

Dimensionality reduction was applied to assess the separability of a different region of interest (ROI) types. Principal component analysis (PCA) based on singular value decomposition was performed on the dataset containing all 1536 components. To account for significant differences in variable ranges without losing information on feature covariance, the data were scaled using a pseudo-logarithmic function (log_10_(x + 1)) and centered. A normalized dot-product of two mass spectra (pairwise similarity index) was calculated to assess the similarity between the compared pair of spectra [40]. Spectra were labeled according to their tissue microarray core location in one of seven ROIs creating seven spectra subsets. The similarity index was calculated in two manners: within particular ROI (intra-ROI similarity) and between different ROIs (inter-ROI similarity) creating all possible combinations of compared spectra pairs. Populations of computed similarity values were plotted as cumulative distribution functions (CDFs) to visualize similarities within and between analyzed ROIs. Sample classification involved two types of classifiers: binary classifiers used to classify individual spectra and the main core classifier. In the first stage, the dataset was standardized using the Z-score method, then support vector machines (SVM) classifiers with a linear kernel were used with a one-versus-one strategy (21 binary classifiers were tested to cover all possible pairs of seven ROIs). An adapted k-fold method was used for model cross-validation. Each patient was assigned to one of the seven classes based on the ROI type identified in the majority of tissue cores derived from that donor. The patients were then partitioned into five stratified folds. The training and validation sets for each fold were reconstructed from this partition, preserving original tissue type of the cores. Such subsampling of the spectra, when quantitatively unbalanced (contrary to the classic k-fold), ensures independence between folds, preventing data leakage and quality estimation bias. In the second step, the output of the binary classifiers was used to classify the whole tissue core. For each spectrum, all 21 binary classifications were tested and a spectrum was considered classified if the decision was univocal, i.e., all six models featuring a particular type indicated that class (otherwise, the spectrum remained not classified). The final classifier decision for the core was the most frequent of eight classes (seven tissue types ROI or “not diagnostic” if the majority of spectra could not be conclusively classified). Basic classification quality indices were calculated using the one-versus-rest approach. An effect size analysis was applied to indicate discriminatory molecular components. Cohen’s *d* value defined here as the difference between the mean abundance of each molecular component between different ROIs divided by pooled standard deviation was calculated [26]. Unlike the t-test statistic, the Cohen’s *d* is independent of the sample size, thus avoiding overestimation of the significance of differences between extremely numerous samples (which is typical for MSI data analysis, where large spectra collections are compared); the Cohen’s *d* absolute value above 0.5, 0.8, and 1.2 corresponds to medium, large, and very large effects, respectively.

### 4.6. LC-MALDI MS/MS Analysis and Identification of Molecular Components

Representative samples of the cancerous thyroid gland (ca. 60% of cancer cells, FFPE material) were used for protein identification using the shotgun LC-MS/MS approach. Protein lysates were prepared and subjected to tryptic digestion according to a modified version of a combination of FASP with stage-tip fractionation as described in detail elsewhere [27]. Tryptic peptides were then separated using an EASY-nLC nano-liquid chromatography coupled with PROTEINEER fcII fraction collector (Bruker Daltonik, Bremen, Germany) and analyzed using an ultrafleXtreme mass spectrometer (Bruker Daltonik, Bremen, Germany). A detailed description of the instrumental settings of the LC-MALDI-MS/MS system is given in [27]. Registered MS/MS spectra were exported to ProteinScape 3.1 software (Bruker Daltonik, Bremen, Germany) and analyzed using Mascot Server 2.5.1 (Matrix Science, London, UK); for details, see Gawin et al. [27]. The hypothetical identity of molecular components detected by MSI was determined by the assignment of the mean parameters of MSI components (component location on mass/charge scale) to the measured masses of tryptic peptides identified in LC-MS/MS experiment; the assignment was performed allowing ±0.05% mass tolerance. The MSI molecular component annotations were established based on peptides identified in three major types of thyroid cancer (ATC, MTC, and well-differentiated thyroid cancers).

## 5. Conclusions

Molecular diagnostics of thyroid disease using the fine-needle cytological biopsy represent a general challenge for “classical” methods of proteomics. This problem could be overcome by mass spectrometry imaging, where sample classification is possible based on a single spectrum that corresponds to small clusters of cells. This approach was recently validated by Capitoli and colleagues who implemented the so-called pixel-by-pixel approach for the bi-state discrimination of malignant and hyperplastic (benign) thyroid nodules using actual diagnostic FNA material analyzed by MALDI-TOF-MSI [19]. Here, we confirmed that a similar approach could be implemented in a more complex situation when multiple classes are to be compared and classified. Our classification model cannot be implemented directly for the analysis of cytological material due to structural differences between MS spectra registered for tissue microarrays based on FFPE and fresh material in actual FNA. However, the proposed single-pixel approach to multi-state classification problems has potentially general applicability not limited to thyroid tumors.

## Supplementary Materials

The following are available online at https://www.mdpi.com/1422-0067/21/17/6289/s1. Figure S1. Average spectra of each type of region of interest (ROI).; Table S1. Included tissue specimens.; Table S2. Intra-ROI similarity of spectral features.; Table S3. Inter-ROI similarity of spectral features.; Table S4. Indices of multicomponent classifiers.; Table S5. Significance of differences in the abundance of molecular components detected by MSI between tissue types shown as the Cohen’s *d* effect size.; Table S6. Hypothetical identity of peptide components detected by MSI based on peptides identified in well-differentiated thyroid cancers.; Table S7. Hypothetical identity of peptide components detected by MSI based on peptides identified in medullary thyroid cancers.; Table S8. Hypothetical identity of peptide components detected by mass spectrometry imaging (MSI) based on peptides identified in anaplastic thyroid cancers.; Table S9. Gene ontology (GO) terms associated with proteins that were hypothetically annotated with MSI components upregulated in specific cancer ROIs.

## Abbreviations

ATC	anaplastic thyroid carcinoma
FA	follicular adenoma
FFPE FTC	formalin-fixed paraffin-embedded follicular thyroid carcinoma
KNN	k-nearest neighbors
MALDI-TOF MS	matrix-assisted laser-desorption ionization time-of-flight mass spectrometry
MSI	mass spectrometry imaging
MTC	medullary thyroid carcinoma
NT	normal thyroid
PTC-CV	papillary thyroid carcinoma–classical variant
PTC-FV	papillary thyroid carcinoma–follicular variant
SVM ROI	support vector machine region of interest
TMA	tissue microarrays
